# The treatment of bacterial biofilms cultivated on knee arthroplasty implants using the bioelectric effect

**DOI:** 10.3389/fbioe.2024.1426388

**Published:** 2024-07-02

**Authors:** Iskandar Tamimi, María Gasca, Alexandra Halbardier, Sergio Martin, Gregorio Martin Caballero, Cristina Lucena Serrano, Elena Martin, Faleh Tamimi, David González-Quevedo, David García de Quevedo, Beatriz Sobrino, Begoña Palop, Enrique Guerado, Almudena Pérez Lara, Cristina Urdiales, Jesús Manuel Gómez de Gabriel

**Affiliations:** ^1^ Orthopedic Surgery Department, Regional University Hospital of Malaga, Málaga, Spain; ^2^ Hospital HM de Malaga, Málaga, Spain; ^3^ Faculty of Medicine, University of Malaga, Málaga, Spain; ^4^ Malaga Institute of Biomedical Research IBIMA, Málaga, Spain; ^5^ Microbiology Department, Regional University Hospital of Malaga, Málaga, Spain; ^6^ Microscopy Service. Central Research Support Services, University of Malaga, Málaga, Spain; ^7^ Faculty of Oral Health, University of Doha, Doha, Qatar; ^8^ Infectious Diseases Department, Regional University Hospital of Malaga, Málaga, Spain; ^9^ Radiology Department, Regional University Hospital of Malaga, Málaga, Spain; ^10^ Industrial Engineering School, University of Malaga, Málaga, Spain

**Keywords:** biofilm, bioelectric effect, periprosthetic infection, total knee replacement, *Staphylococcus aureus*

## Abstract

**Introduction:** The formation of bacterial biofilms on knee arthroplasty implants can have catastrophic consequences. The aim of this study was to analyze the effectiveness of the bioelectric effect in the elimination of bacterial biofilms on cultivated knee arthroplasty implants.

**Methods:** A novel device was designed to deliver a bioelectric effect on the surface of knee arthroplasty implants. 4-femoral prosthetic implants were cultivated with a *staphylococcus aureus* inoculum for 15 days. The components were divided into four different groups: A (not treated), B (normal saline 20-minutes), C (bioelectric effect 10-minutes), D (bioelectric effect 20-minutes). The implants were sonicated, and the detached colonies were quantified as the number of colony-forming unit (CFUs). The implants were sterilised and the process was repeated in a standardized manner four more times, to obtain a total of five samples per group.

**Results:** The number of the CFUs after a 10-minute exposure to the bioelectric effect was of 208.2 ± 240.4, compared with 6,041.6 ± 2010.7 CFUs in group A, representing a decrease of 96.5% ± 4.3 (*p* = 0.004). And a diminution of 91.8% ± 7.9 compared with 2,051.0 ± 1,364.0 CFUs in group B (*p* = 0.109). The number of bacterial colonies after a 20-minute exposure to the bioelectric effect was 70 ± 126.7 CFUs, representing a decrease of 98.9% ± 1.9 (*p* = 0.000) compared with group A. And a decrease of 97.8% ± 3.0 (*p* = 0.019) compared with group B.

**Conclusions:** The bioelectric effect was effective in the elimination of bacterial biofilm from knee arthroplasty implants. This method could be used in the future as part of conventional surgical procedures.

## Introduction

The formation of bacterial biofilms on the surface of prosthetic implants can have catastrophic consequences. These bacterial membranes favor the chronification of prosthetic infections, and act as a protective barrier that limits the penetration of antibiotics into the bacterial colonies, which are adhered to the implants (Høiby et al., 2011). On the other hand, the bacterial colonies within the biofilm membrane can be enter a hibernating state for prolonged periods (Khaova et al., 2022). This means that conventional cultures in these cases are frequently negative, which makes the diagnosis of these infections even more difficult. The sonication of the infected implants has been a significant advance in the diagnosis of these infections, that combined with histological sampling, can achieve a sensitivity of 94% (Janz et al., 2015).

The treatment of peri-prosthetic infections in orthopedic surgery is usually long and complex. In addition, it significantly impacts the patients’ physical and psychological state and has an extremely high economic cost (Hernández-Vaquero et al., 2013; Svensson et al., 2020). The latest estimates suggest that the number of hip and knee prosthetic infections will be around 66,000 infections/year in the United States by 2030. This would translate into a cost of 1.85$ billion annually in the United States alone (Premkumar et al., 2021). Moreover, despite the best efforts, a large number of these patients do not overcome the infection. And the 5-year mortality rates after a prosthetic infection can be as high as 25.9%, compared to 12.0% in patients with uninfected primary prostheses (Zmistowski et al., 2013). Furthermore, the prolonged use of antibiotics in the treatment of these infections can create multi-resistant microorganisms that make their eradication even more difficult (Rudelli et al., 2020). Therefore, we can see that there is a need for the development of new therapeutic strategies for the treatment of bacterial biofilms in periprosthetic infections.

On the other hand, there has been growing evidence proposing that the utilization of electrical currents could possibly destroy organized biofilm colonies (DEL et al., 2008; Pareilleux and Sicard, 1970). This phenomenon has been described as the bioelectric effect. Many hypothetical mechanisms have been proposed to explain the bioelectric effect such as: the reduction of biofilm capacity for binding antimicrobial agents (Blenkinsopp et al., 1992); increased membrane permeabilization (Khoury et al., 1992); electrophoretic augmentation of antimicrobial transport (Khoury et al., 1992); increased bacterial growth due to electrolytic generation of oxygen (and subsequently enhanced susceptibility to antimicrobials) (Jass and Lappin-Scott, 1996; Stewart et al., 1999); electrochemical generation of potentiating oxidants (Costerton et al., 1994); and increased convective transport due to contraction and expansion of the biofilms (Stoodley et al., 1997). However, bioelectric effect has not yet been applied in the in treatment of infected prosthetic joint implants.

We hypothesize that the bioelectric effect could be used to eliminate bacterial biofilms from infected arthroplasty implant. A novel bioelectric device was designed to test this hypothesis.

## Material and methods

Ethical approval was not required for this submission.

### Design of the bioelectric device

#### Electrode matrix

The flexible printed circuits were fabricated by PCBWay, Inc. (www.pcbway.com, China). They consisted of a set of silver electrodes (electrode array), which were placed around the external surface of the prosthesis, in a parallel position, maintaining a 2 mm distance with the implant, to avoid direct contact between the electrodes and the prosthesis. In addition, one reference electrode was plugged directly to the side of the implant.

**FIGURE 1:**
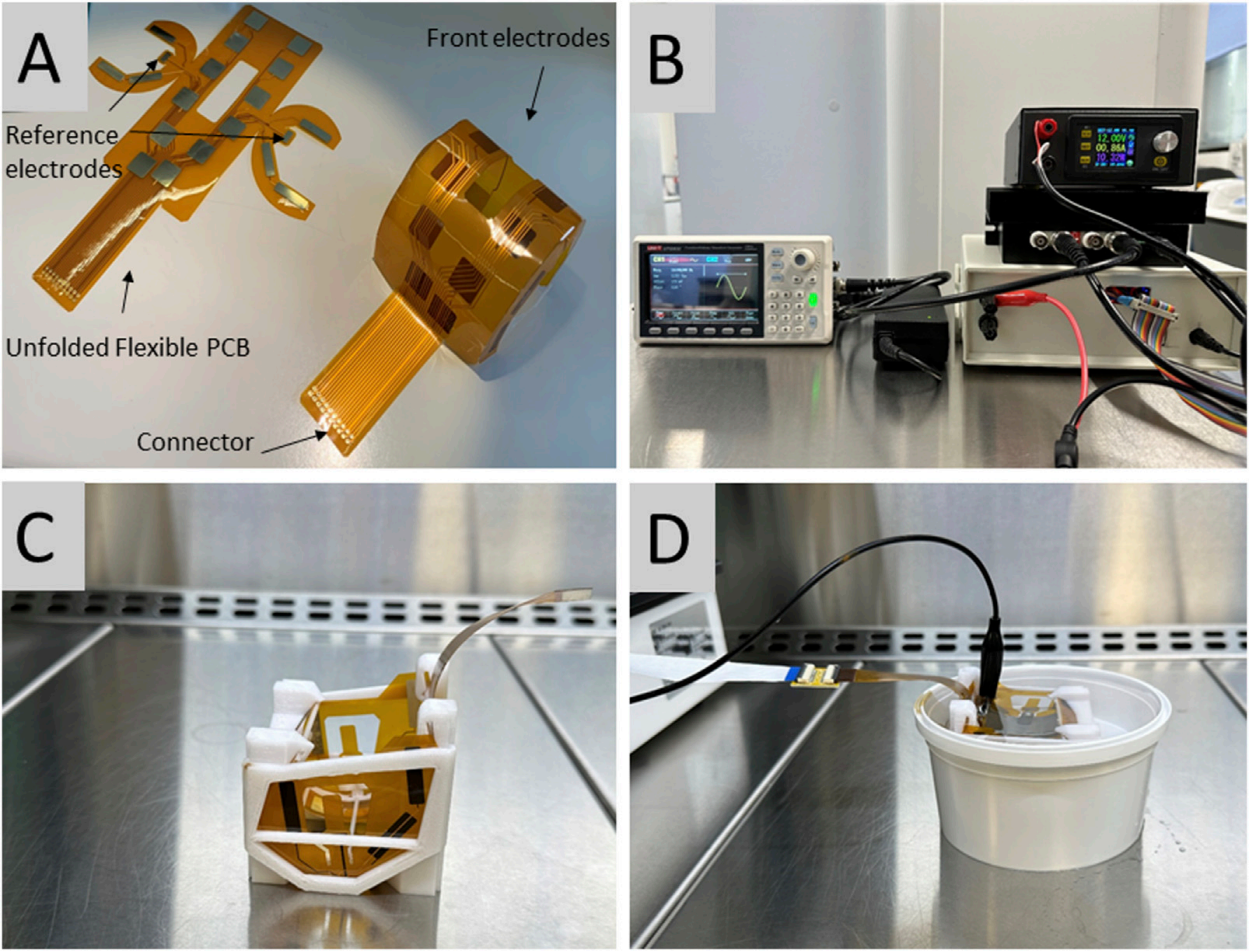
**(A)** The control system that regulates the activation of the different electrodes. **(B)** Electrode matrix consisting of a set of silver electrodes placed around the external surface of the prosthesis. **(C)** The electrode matrix connected to the femoral prosthetic component in a normal saline solution. **(D)** The 3D printed support which secures the position of the matrix on the prosthesis.

The electrodes and the prosthesis were then immersed in a normal saline (NS) solution which served as a conducting medium (Figure 1). An electric current was then generated between the activated electrodes, and transmitted over the surface of the metallic implant. In addition, a control system was created to regulate the activation sequence of the different electrodes to ensure that the current was equally distributed over the surface of the implant. The experiments were conducted with a set of 16 electrodes. The number and distribution of the electrodes determined the area exposed to the electrical current on the surface of the implants (Figure 1).

The electrode matrix was designed using Flexible Printed Circuits (FPCs). These were then bent and assembled to build a light cover adapted to the surface of the implant. The electrodes were silver-plated with copper terminals, and their connections converged in a terminal band that maintained the connector away from the sterile area. There were 12 square electrodes on the front of the matrix measuring 12 × 12 mm, and six rectangular electrodes on the side of the matrix measuring 20 × 5 mm. FPCs resist high temperatures and sanitizing chemicals, and were approved by the U.S. Food and Drug Administration (FDA) for human use.

The position of the matrix of electrodes on the implants was maintained by a 3D printed support made of a synthetic resin that was also approved by the FDA for human use. This support maintained the 2 mm distance between the electrodes and the prosthesis to avoid direct contact (Figure 1).

### The current controller

**FIGURE 2:**
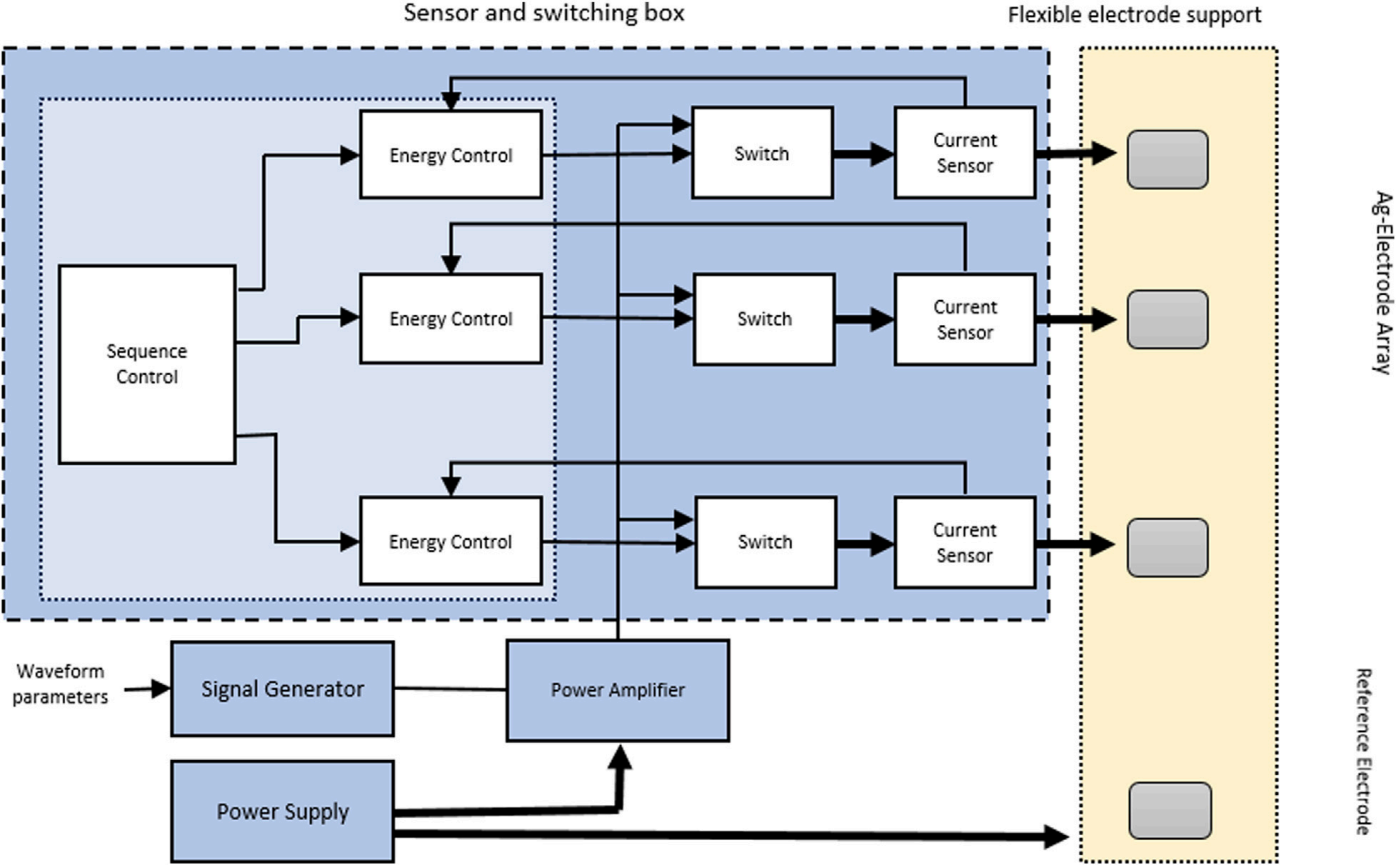
Electronic pathways of the bioelectric device.

The electronic system shown in (Figure 1) was designed to apply energy through the electrodes in a controlled and independent manner, as described in (Figure 2). In this experiment, all the electrodes were activated simultaneously; however, the current controlled was also able to deliver the electrical current on independent electrodes. The system had a single programmable signal generator and power supply, and a current monitor, to power all the electrodes in a synchronized manner. The average current generated between the activated electrodes was of 0.430 A at 12 V (5.28 W).

The electronic system had a programmable function, which was capable of generating different signal waveforms. In this experiment, a sinusoidal waveform with a frequency of 10.000 Hz, an amplitude of 4.5 V, and an offset of 750 mV was used. The resulting alternating signal changed from positive to negative voltages (from −1.5 V to +3.0 V). This range was found not to cause damage to osteoblasts in an unpublished pilot *in vitro* study performed by our team. A linear amplifier with a capacity of 50 W (FPA2000 from www.feeletec.com) was connected to all the electrodes, and the reference signal was connected to the reference electrodes. Bipolar electrodes were used because they can act as an electronic conductor in contact with an ionically conductive phase. When a sufficient voltage was applied, it experienced simultaneous cathodic and anodic reactions at both extremes. The reference electrodes were two small electrodes located on the sides of the matrix measuring 8 × 5 mm, which were bent to make direct contact with the femoral implant (Figure 1). The signal was fed into a linear analog power amplifier. Both the implant and the device were submerged in NS 0.9%. The solution acted as a transmission medium, and currents from 1.3 to 1.9 A (15.6–22.8 W) were obtained. The conduction of NS 0.9% has been described to be of 1.45 S/m (Falco et al., 2022).

No pulse wave modulation (PWM) or digital switching methods were used to provide a true analogic signals. The “Device Control” and “Energy Control” blocks were managed by using a software in a microcontroller, while the rest of the blocks were physical components.

### Culture of the bacterial biofilm

The culture of the bacterial biofilm on the prosthetic component followed a protocol previously described in the literature (Marques et al., 2007). We used not-previously used four smooth-surface Sigma size two femoral prosthetic components for knee arthroplasty (DePuy Synthes, Raynham, MA, United States) (Figure 1). These implants are made of a chrome-cobalt alley (McEwen et al., 2005). The implants were immersed in a solution of 60 mL of ¨Brain heart infusion¨ (BHI) and 10 mL of an inoculum of *Staphylococcus aureus* ATCC 25923 and incubated at a temperature of 37°C for 72 h. The implants were then washed with a sterile phosphate buffered saline solution (PBS, pH 7.4) to remove unattached cells. The prostheses were then immersed again in a new solution equal to the one previously used with the same amount of inoculum. The washes were carried out every 72 h, and the process was repeated 4 times to complete a total of 15 days (Marques et al., 2007).

### Exposure to the bioelectrical device

The prosthesis were divided into four different groups: group A (no further treatment), group B (NS bath, and plugged to the bioelectric device for 20 min; no electrical current was applied), group C (NS bath, and plugged to the bioelectric device; electrical current applied for 10 min), group D (NS bath, and plugged to the bioelectric device; electrical current applied for 20 min).

### Quantification of the bacterial colonies

The implants underwent an ultrasound sonication treatment using a USC-T ¨Ultrasonic-cleaner¨ device (VWR, Radnor, Pennsylvania, United States). The detached bacteria were then cultured overnight, and the number of colony-forming units (CFUs) was quantified using ImageJ software (National Institutes of Health, Bethesda, Maryland). The prostheses were then subjected to autoclave sterilization and the whole process was repeated in a five cycles to include five samples in each group (*n* = 20). All steps in the laboratory were standardized under the same conditions.

### Electronic microscope

Two additional implants were cultivated (one was treated as in group B and the second as in group D). These were then fixed with 2% glutaraldehyde in 0.1 M Cacodylate buffer at pH = 7.4, for 1 h at room temperature. Then they were washed 3 × 10 min with Cacodylate Buffer + Iso-osmolar Sucrose with the Postfix fixer with 1% OsO4 in Cacodylate, for 1 h in the dark at four°C. Next, they were washed 3 times with Cacodylate, 10 min and dehydrated in increasing series of EtOH (30%, 50, 70, 90, 96, 100 and 2 times 100%). Finally, the implants were washes two more times with hexamethyldisilane for 10 min and then were left to dry.

Once fixed, they were placed on a scanning electron microscope support using conductive cement. Metallic coating with gold was carried out in an Argon atmosphere and the surface was visualized with a scanning electron microscope Jeol JSM-6490LV (Akishima, Tokyo 196–8558, Japan).

### Data analyses

**FIGURE 3:**
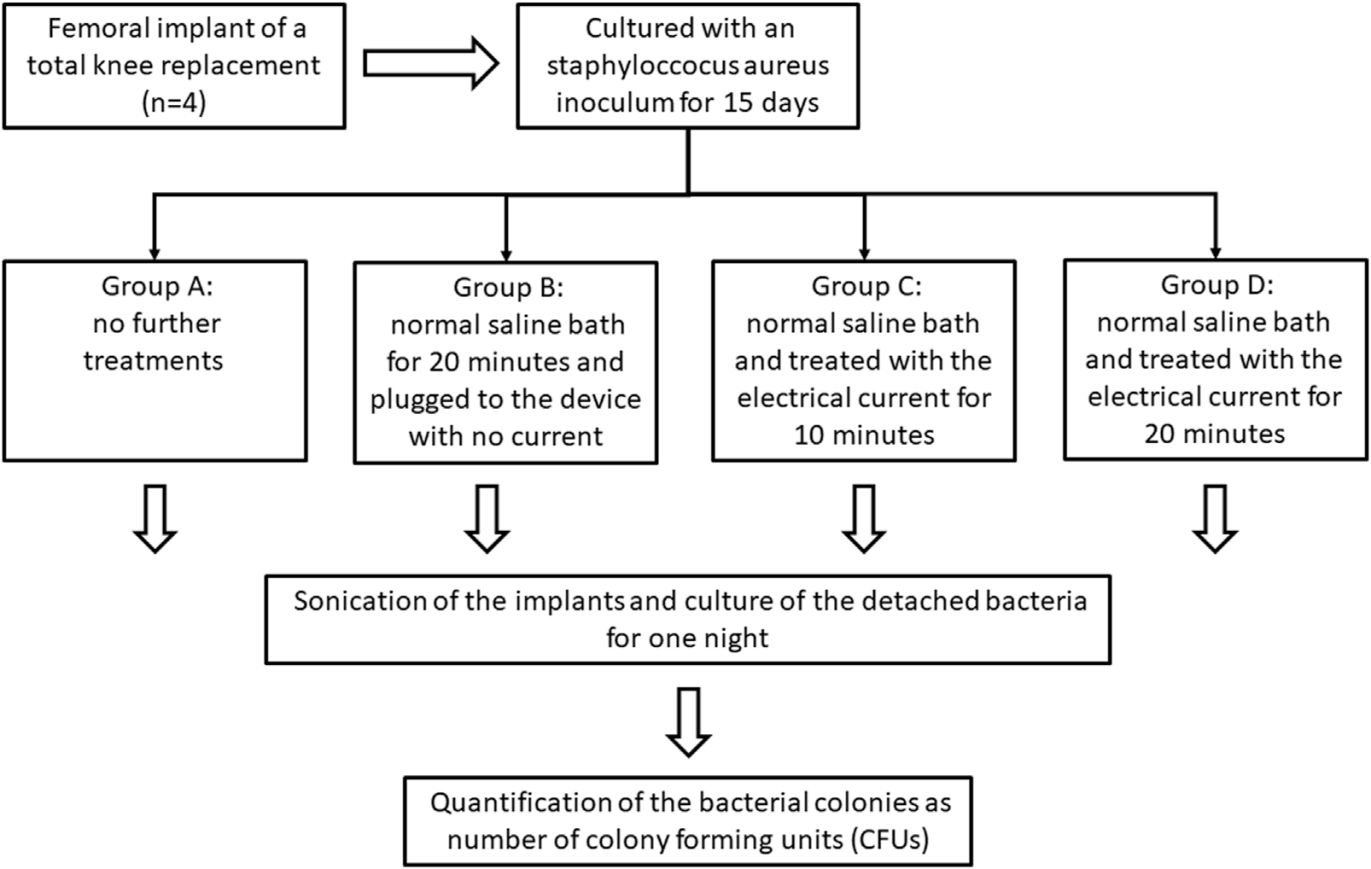
Scheme showing the steps of the experiment (one cycle), including the culture of the prosthesis, the separation of the different groups, the sonication of the implants, and the quantification of the colony-forming units (CFUs). This step was repeated 5 times.

The statistical analysis was performed using the statistical analysis system IBM SPSS Statistics 20.0 (IBM Inc., Chicago, IL, United States). A Shapiro-Wilk test was performed on continuous variables. A Kruskal-Wallis test for independent samples was performed to analyse significant differences between groups. *p*-values were considered statistically significant if less than 0.05. A retrospective *post hoc* power analysis for independent samples was performed using G* Power software (University of Dusseldorf, Germany) (Jacob, 2013). The dataset used for the statistical analysis is available as supplementary information. The flow chart presented in (Figure 3) summarizes the experimental steps of the sections below.

## Results

**FIGURE 4:**
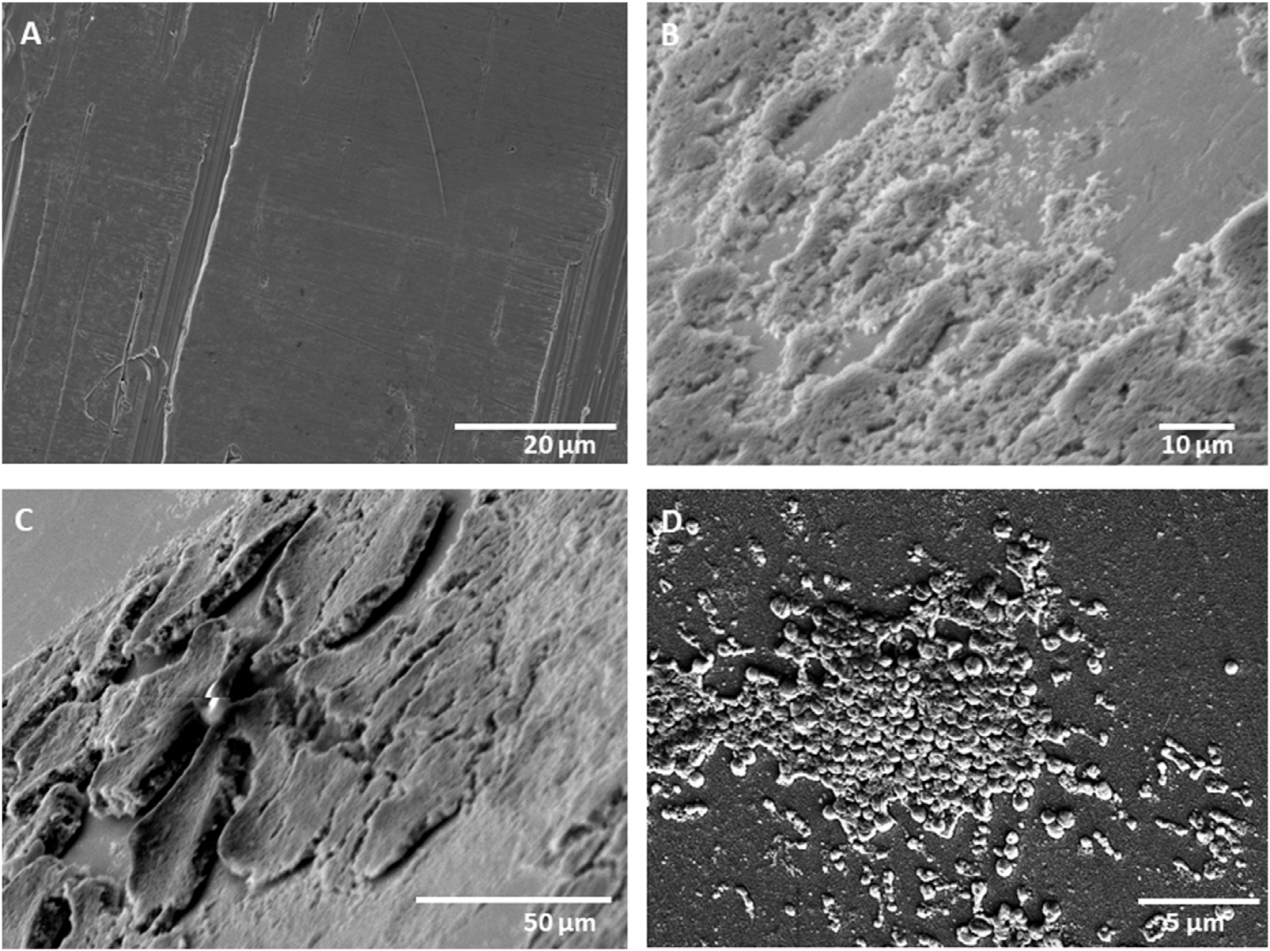
**(A)** Electronic microscope imaging showing a clean metallic surface before the growth of the bacterial biofilm. **(B,C)** The mature bacterial biofilm at 1000 and 500 magnifications, respectively. **(D)** The growth cocci colonies on the metallic surface at 5000 magnifications.

**FIGURE 5:**
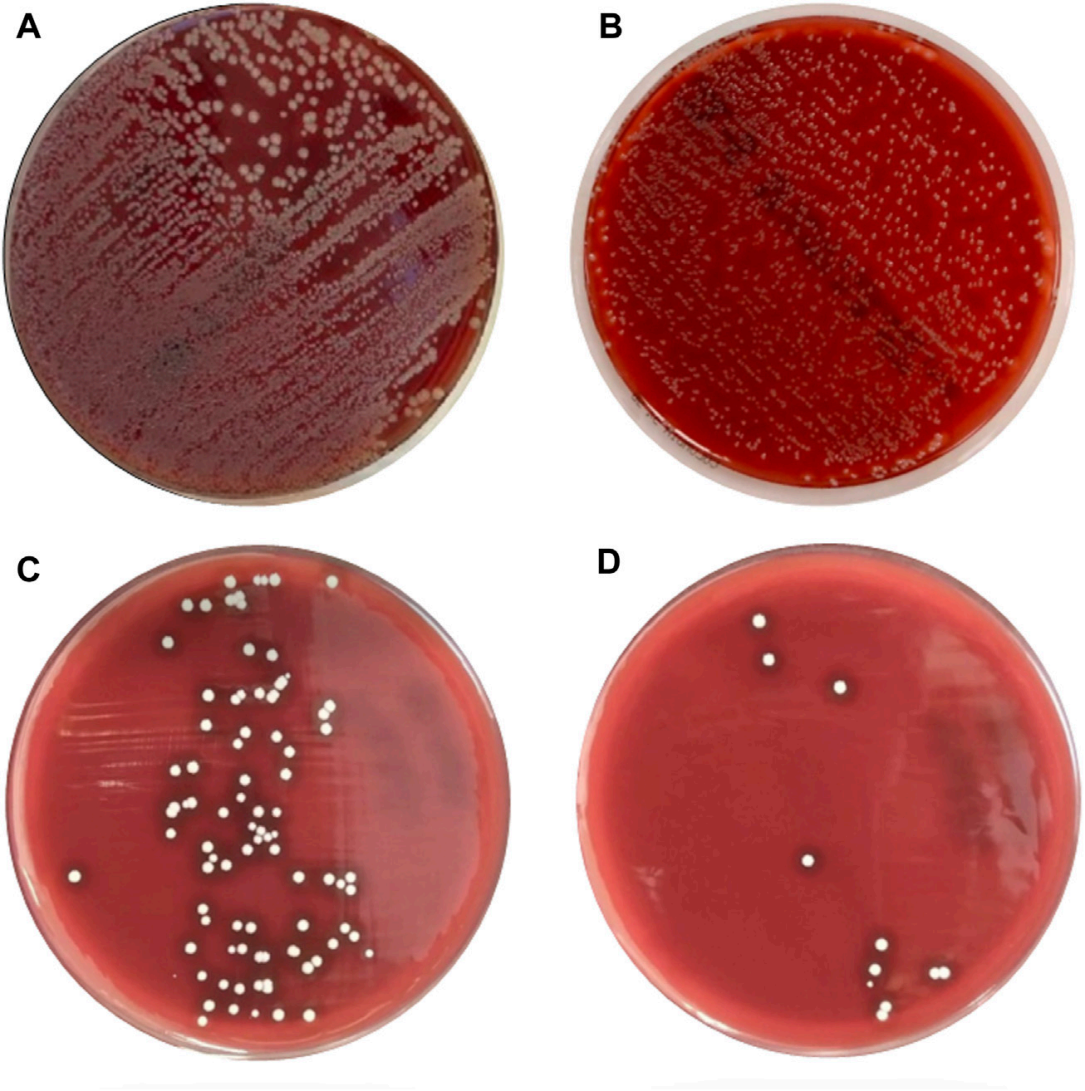
Growth the of staphylococcus aureus colonies after a 15 day period of biofilm culture on the prosthetic components. **(A)** After direct sonication; **(B)** After a 20 min wash in normal saline; **(C)** after treatment with the bioelectric device for 10 min; **(D)** after treatment with the bioelectric device for 20 min.

**FIGURE 6:**
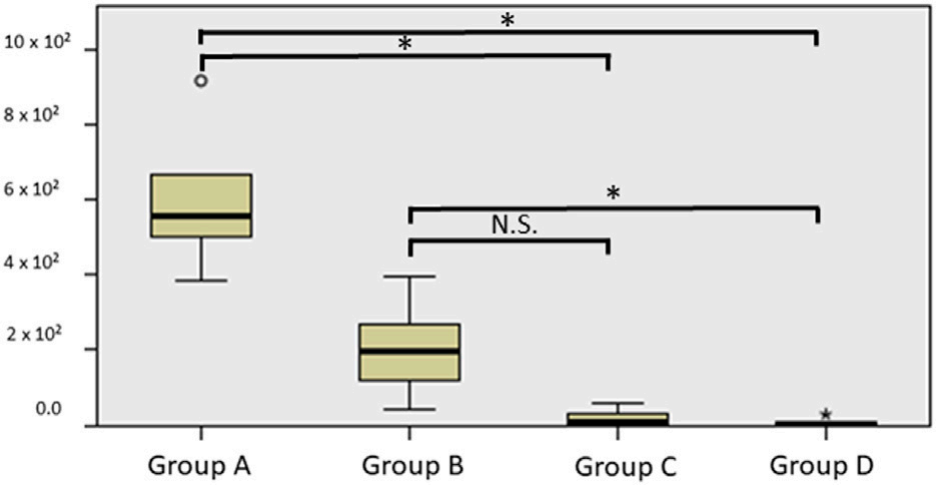
Boxplot showing the colony forming units (CFUs) in group A. group A (direct sonication), group B (immersed in normal saline, and plugged to the device with no current for 20 min), group C (immersed in normal saline, plugged and treated with the electrical current for 10 min), group D (immersed in normal saline, plugged and treated with the electrical current for 20 min).

The growth of the staphylococcal biofilm on the metallic prosthesis was confirmed by the electrical microscope (Figure 4). The total number of the CFUs after a 10-min exposure to bioelectric effect was of 208.2 ± 240.4 that is a diminution of 5,833.4 ± 2,009 CFUs compared with 6,041.6 ± 2010.7 CFUs in group A, representing a decrease of 96.5% ± 4.3 (*p* = 0.004). And a diminution of 1,842.8 ± 1,209.6 CFUs compared with 2,051.0 ± 1,364.0 CFUs in group B, representing a decrease of 91.8% ± 7.9 (*p* = 0.109) (Tables 1–3; Figures 5, 6). When comparing group, A 6,041 ± 2,010.7 CFUs and group B 2,051 ± 1,364.0 CFUs, the reduction was of 3,990.0 ± 2238.3 CFUs of 65.6% ± 21.6 (*p* = 0.016) (Tables 1–3 and Figures 5, 6).

**TABLE 1:** The number of CFUs grown after sonication of each group.

	Group A (*n* = 5)	Group B (*n* = 5)	Group C (*n* = 5)	Group D (*n* = 5)
Mean CFUs, SD	6041.6 ± 2010.7	2051.0 ± 1364.0	208.2 ± 240.4	70.0 ± 126.7

Abbreviation: SD, standard deviation; number of forming colonies, CFUs; normal saline, NS.

Group definition: group A (no further treatment), group B (NS, bath, and plugged to the bioelectric device for 20 min; no electrical current was applied), group C (NS, bath, and plugged to the bioelectric device; electrical current applied for 10 min), group D (NS, bath, and plugged to the bioelectric device; electrical current applied for 20 min).

**TABLE 2:** The reduction in the CFUs after a 10 and 20 min exposure to the bioelectric device (Group C vs. A and B; and Group D vs. A and B).

	Group C vs. Group A	Reduction percentage (%)	Group C vs. group B	Reduction percentage (%)
Mean reduction, SD	5833.4 ± 2009.0	96.5 ± 4.3	1842.8 ± 1209.6	91.8 ± 7.9
	Group D vs. Group A	Reduction percentage	Group D vs. group B	Reduction percentage
Mean reduction, SD	5971.6 ± 1987.2	98.9 ± 1.9	1981.0 ± 1265.4	97.8 ± 3.0

Abbreviation: SD, standard deviation; number of forming colonies, CFUs; normal saline, NS.

Group definition: group A (no further treatment), group B (NS, bath, and plugged to the bioelectric device for 20 min; no electrical current was applied), group C (NS, bath, and plugged to the bioelectric device; electrical current applied for 10 min), group D (NS, bath, and plugged to the bioelectric device; electrical current applied for 20 min).

**TABLE 3:** The effect of the bioelectric device on the CFUs.

Compared groups	CFUs reduction	Reduction percentage (%)	*p*-value	Statistical power (%)
Group A vs. Group C	5833.4 ± 2009	96.5 ± 4.3	0.004*	100.0
Group B vs. Group C	1842.8 ± 1209.6	91.8 ± 7.9	0.109	74.1
Group A vs. Group D	5971.6 ± 1987.2	98.9 ± 1.9	0.000*	100.0
Group B vs. Group D	1981.0 ± 1265.4	97.8 ± 3.0	0.019*	80.8

*Statistically significant.

Abbreviation: SD, standard deviation; number of forming colonies, CFUs; normal saline, NS.

Group definition: group A (no further treatment), group B (NS, bath, and plugged to the bioelectric device for 20 min; no electrical current was applied), group C (NS, bath, and plugged to the bioelectric device; electrical current applied for 10 min), group D (NS, bath, and plugged to the bioelectric device; electrical current applied for 20 min).

On the other hand, the total number of the CFUs after a 20-min exposure to bioelectric effect was of 70 ± 126.7 that is a diminution of 5,971.6 ± 1,987.2 CFUs compared with 6,041.6 ± 2,010.7 CFUs in group A, which represent a decrease of 98.9% ± 1.9 (*p* = 0.000). And a diminution of 1,981.0 ± 1,265.4 CFUs compared with 2,051.0 ± 1,364.0 CFUs in group B, representing a decrease of 97.8% ± 3.0 (*p* = value 0.019) (Tables 1–3 and Figures 5, 6).

## Discussion

Several strategies have been followed in order to prevent periprosthetic infections, such as the use of antibiotic-impregnated cements, intraoperative irrigation, or the use of laminar flow systems (Daines et al., 2015). However, despite all these precautions, the incidence of these infections is around 1%–2% in the case of primary TKA and 0.5–1.5 in total hip arthroplasty (THA) (Kong et al., 2017; Izakovicova et al., 2019). Some studies have estimated that the economic burden of periprosthetic hip and knee infections will reach 1.85 billion dollars per year by 2030 in the United States alone. Moreover, it is estimated that these figures will continue to increase in the coming years due to the aging world population (Premkumar et al., 2021).

Periprosthetic joint infections have been traditionally treated following three different strategies, depending on the onset and severity of the infection: debridement with retention of the implant (DAIR), single-stage revision, and two-stage revision surgery (Li et al., 2018). A DAIR, which is the least invasive option, consists in the removal of any mobile components (e.g., polyethylene surface) and thorough debridement and irrigation of the joint. The patient is then treated with antibiotics for a prolonged period of time (Scheper et al., 2021). In a recent meta-analysis focused on the use of rifampicin, DAIR, achieved a success rate of 69% and 54% in the treatment of THA and TKA staphylococcus aureus infections, respectively. Other studies have reported success rates of 50%–70% in cases of early infections after revision surgeries (Hulleman et al., 2023). Single-stage revision consists in the removal of all the infected components and their replacement with new implants in the same surgical act (Li et al., 2018); whereas in a two-stage revision, the implantation of the new prosthesis is delayed (Li et al., 2018). A recent meta-analysis performed on retrospective and prospective cohort studies, reported 7.6% reinfection rates following a single-stage revision compared with 8.8% in two-stage revisions (Kunutsor et al., 2016). Nevertheless, periprosthetic joint infections are associated with high mortality rates, similar to the 5-year mortality rates of the five most common cancers. When comparing the 5-year mortality rates of revision TKA for aseptic loosening vs. revision of an infected TKA, the latter is 5–6 times higher (Drain et al., 2022).

On the other hand, there has been growing evidence proposing that the utilization of an electrical current could possibly destroy organized biofilm colonies (DEL et al., 2008; Pareilleux and Sicard, 1970). Previous, *in-vitro* studies have shown that direct electric current densities and electric fields could enhance the activities of certain biocides and antimicrobial agents; this has been named the “bioelectric effect”. The antibacterial activity of the electric current has been previously demonstrated against *Escherichia coli* in salt solutions (Pareilleux and Sicard, 1970), *Staphylococcus aureus* in agar (Barranco et al., 1974), normal flora on human skin (Bolton et al., 1980), *Escherichia coli*, *Proteus* species and *Klebsiella pneumoniae* in synthetic urine (Davis et al., 1991), and *E. coli*, *Staphylococcus aureus* and *Bacillus subtilis* in water (Matsunaga et al., 1992). In this study, we selected *Staphylococcus aureus* for biofilm cultivation because it is the most common bacteria isolated in periprosthetic infections, and contributes to approximately 50%–60% of all the periprostheric infections (Tande and Patel, 2014). Our novel device has shown that the bioelectric effect could be effectively used to destroy staphylococcal aureus biofilms from the surface of chrome-cobalt orthopaedic implants. Moreover, we observed that the best results were observed following a 20-min exposure to the electrical current achieving a 97.8% ± 3.0 reduction in the total number of bacterial colonies compared with 91.8% ± 7.9 after a 10 min exposure. These results are in consonance with previous studies that have shown that the effectiveness of the bioelectric effect could increase with the time of exposure. However, it is still unclear which parameters are more relevant (e.g., voltage, current intensity, time of application) (DEL et al., 2008).

The antibacterial mechanism of the action of the bioelectric effect has been attributed to the release of toxic substances as a result of electrolysis (e.g., H_2_O_2_, oxidizing radicals, chlorine molecules), oxidation of enzymes and coenzymes, membrane damage prompting to leakage of basic cytoplasmic constituents, and/or diminished bacterial respiratory rate (Davis et al., 1994). According to several studies, the efficacy of biocides (Blenkinsopp et al., 1992) and antibiotics (Khoury et al., 1992) in killing biofilm bacteria can be radically improved and be more effective if these antibiotics are used within a low-intensity electric field. Costerton et al., showed in 1994 (Costerton et al., 1994) that the efficiency of certain antimicrobial agents could be expanded through the use of weak electric fields. The authors observed that with the combined application of direct current electric fields of about 1.5–20 V/cm2 (current densities of about 15 × 10^−6^ to 2.1 × 10^−3^ A/cm^2^) and tobramycin, the concentration of the antimicrobials needed to exhibit activity against the biofilm bacteria fell by 1.5–4.0 times, compared to that needed against planktonic bacteria. Moreover, Jass et al., demonstrated that an electrical current could enhance the activity of some antimicrobials (i.e., ciprofloxacin and polymyxin B), but not of others such as piperacillin against *Pseudomonas aeruginosa* (Jass and Lappin-Scott, 1996).

On the other hand, the application of the bioelectric effect in a surgical setting should take in consideration several safety factors, such as changes in temperature, potential cellular damage, and its effect on the bone-implant interphase. The dynamic response of bone cells to mechanical and electrical changes is necessary to induce the production of growth factors, intracellular calcium, and bone remodeling. Moreover, electrical stimulation devices have been used in the past to induce fracture healing (Isaacson and Bloebaum, 2010). On the other hand, recent research has shown that the bioelectric effect was able to completely eradicate the bacterial biofilm from saliva-contaminated titanium surfaces within 5 min of exposure without damaging mammalian tissues (Al-Hashedi et al., 2016). In another study, researchers reported that the exposure to 0-hz static and 50-hz electric field may affect bone healing tissue of tibial fractures in rats; however, their results were not significant, and the exposure to the bioelectric effect in this study was of several weeks (Aslan et al., 2020). Therefore, a relatively short exposure to a low electric current is not expected to cause significant effects on the surrounding bone tissues. Nevertheless, research on the safety of the bioelectric effect is scarce and further research should be conducted in the future to add insight in the question.

### Strengths and limitations

To the best of our knowledge this is the first time the bioelectric effect has been used to clean the surface of an intraarticular prosthetic implant, eradicating up to 97.8% ± 3.0 of the bacterial colonies. Our device has been designed for its intraoperative application as part of standard DAIR protocols, and could therefore reduce the need for one stage and–two stage revision surgeries in the future. Our results showed that washing the prosthesis with NS, the method normally used in DAIR, only reduces bacterial colonies by 65.6% ± 21.6. Therefore, the use of the bioelectric effect could substantially improve the results of DAIR, and potentially avoid the need to replace the prosthetic implants in the future.

However, our study is subject to several limitations. Firstly, it only included one type of bacterial cultures, and future studies should analyze its effect on other different common pathogens such as staphylococcus epidermidis, *Escherichia coli* and *Klebsiella* strains. Other electrical currents and exposure times should be studied in the future to determine the optimal combination of variables to achieve the highest effectiveness of the bioelectric device. Moreover, future bioelectrical devices should be adaptable to the anatomy of the knee and surgical requirements. Therefore, further cadaveric and clinical studies should be performed in the future before its inclusion in surgical protocols for the treatment of periprosthetic infections.

## Conclusions

This novel bioelectric device was effective in the elimination of bacterial biofilms from the surface of TKA implants *in vitro*. The bioelectric effect could be used in the future as part of conventional DAIR procedures.

## Data Availability

The original contributions presented in the study are included in the article/supplementary material, further inquiries can be directed to the corresponding author.
