# Supplementary material for: The treatment of bacterial biofilms cultivated on knee arthroplasty implants using the bioelectric effect
Source: Front Bioeng Biotechnol. 2024 Jul 2;12:1426388. doi: 10.3389/fbioe.2024.1426388 (PMC11249753; doi:10.3389/fbioe.2024.1426388)
Supplement: Supplementary file 1 [file DataSheet1.PDF]

Data [Vista protegida] - Excel

ARCHIVO INICIO INSERTAR DISEÑO DE PÁGINA FÓRMULAS DATOS REVISAR VISTA

A1 | fx N_colonies_A_vs_D

	A	B	C	D	E	F	G
1	N_colonies_A_vs_D	N_colonies_B_vs_D	N_colonies_A_vs_C	N_colonies_B_vs_C	N_colonies	Groups_A_B_C_D	
2	3.846	429	3.846	429	3.846	00	
3	5.024	1.966	5.024	1.966	5.024	00	
4	9.156	1.192	9.156	1.192	9.156	00	
5	5.538	2.712	5.538	2.712	5.538	00	
6	6.644	3.956	6.644	3.956	6.644	00	
7	00	00	07	07	429	01	
8	21	21	43	43	1.966	01	
9	22	22	89	89	1.192	01	
10	11	11	576	576	2.712	01	
11	296	296	326	326	3.956	01	
12					07	02	
13					43	02	
14					89	02	
15					576	02	
16					326	02	
17					00	03	
18					21	03	
19					22	03	
20					11	03	
21					296	03	
22							
23							
24							
25							
26							
27							
28							

Data
